# Methyl 5-chloro-2-hydr­oxy-3-(4-methoxy­phen­yl)-4,6-dimethyl­benzoate

**DOI:** 10.1107/S1600536809031614

**Published:** 2009-08-19

**Authors:** Muhammad Adeel, Irshad Ali, Peter Langer, Alexander Villinger

**Affiliations:** a Gomal University, Department of Chemistry, Dera Ismail Khan (NWFP), Pakistan; b Universität Rostock, Institut für Chemie, Abteilung für Anorganische Chemie, Albert-Einstein-Strasse 3a, 18059 Rostock, Germany; c Leibniz-Institut für Katalyse e.V. an der Universität Rostock, Albert-Einstein-Strasse 29a, 18059 Rostock, Germany

## Abstract

In the title compound, C_17_H_17_ClO_4_, the dihedral angle between the mean planes of the two benzene rings is 65.92 (5)°. The methyl ester group lies within the ring plane [deviations of O atoms from the plane = −0.051 (2) and 0.151 (2) Å] due to an intra­molecular O—H⋯O hydrogen bond. In the crystal, mol­ecules are held together by rather weak non-classical inter­molecular C—H⋯O hydrogen bonds, resulting in dimeric units about inversion centers, forming eight- and ten-membered ring systems as *R*_2_^2^(8) and *R*_2_^2^(10) motifs.

## Related literature

For the pharmacological relevance of 3-aryl­salicylates, see: Buchanan *et al.* (1997 ▶); Huang *et al.* (1999 ▶); Lin, Lin & Kuo (1997 ▶); Lin, Wu & Kuo (1997 ▶). For the synthesis, see: Adeel *et al.* (2009 ▶); For hydrogen-bond motifs, see: Bernstein *et al.* (1994 ▶).
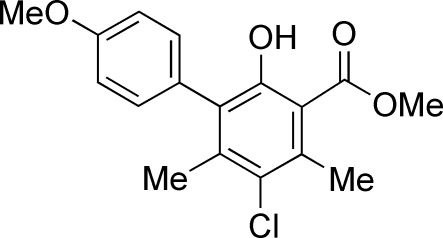


## Experimental

### 

#### Crystal data

C_17_H_17_ClO_4_
*M* *_r_* = 320.76
Triclinic, 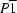

*a* = 6.534 (4) Å
*b* = 9.574 (6) Å
*c* = 12.694 (8) Å
α = 97.420 (15)°
β = 100.56 (2)°
γ = 96.042 (14)°
*V* = 767.3 (8) Å^3^
*Z* = 2
Mo *K*α radiation
μ = 0.26 mm^−1^
*T* = 173 K
0.55 × 0.27 × 0.01 mm

#### Data collection

Bruker APEXII CCD diffractometer
Absorption correction: multi-scan (*SADABS*; Sheldrick, 2004 ▶) *T*_min_ = 0.868, *T*_max_ = 0.997
14947 measured reflections
3964 independent reflections
3091 reflections with *I* > 2σ(*I*)
*R*_int_ = 0.024

#### Refinement

*R*[*F* ^2^ > 2σ(*F* ^2^)] = 0.040
*wR*(*F* ^2^) = 0.125
*S* = 1.09
3964 reflections
207 parameters
H atoms treated by a mixture of independent and constrained refinement
Δρ_max_ = 0.31 e Å^−3^
Δρ_min_ = −0.23 e Å^−3^

### 

Data collection: *APEX2* (Bruker, 2003 ▶); cell refinement: *SAINT* (Bruker, 2003 ▶); data reduction: *SAINT*; program(s) used to solve structure: *SHELXS97* (Sheldrick, 2008 ▶); program(s) used to refine structure: *SHELXL97* (Sheldrick, 2008 ▶); molecular graphics: *ORTEP-3* (Farrugia, 1997 ▶) and *DIAMOND* (Brandenburg, 2005 ▶); software used to prepare material for publication: *SHELXL97*.

## Supplementary Material

Crystal structure: contains datablocks I, global. DOI: 10.1107/S1600536809031614/pv2189sup1.cif

Structure factors: contains datablocks I. DOI: 10.1107/S1600536809031614/pv2189Isup2.hkl

Additional supplementary materials:  crystallographic information; 3D view; checkCIF report

## Figures and Tables

**Table 1:** Hydrogen-bond geometry (Å, °)

*D*—H⋯*A*	*D*—H	H⋯*A*	*D*⋯*A*	*D*—H⋯*A*
O1—H1⋯O2	0.94 (2)	1.63 (2)	2.5061 (18)	153 (2)
C10—H10*A*⋯Cl1	0.98	2.45	3.003 (2)	115
C9—H9*A*⋯O2^i^	0.98	2.73	3.242 (3)	113
C15—H15⋯O4^ii^	0.95	2.50	3.437 (2)	170

Symmetry codes: (i) 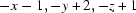; (ii) 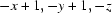.

**Fig. 1.**
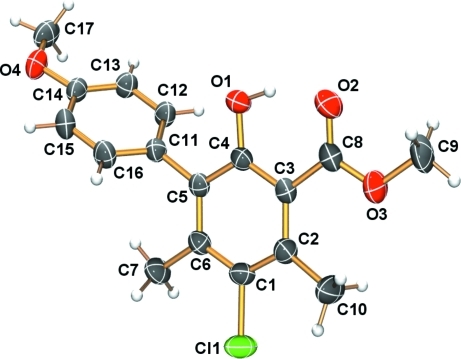
Molecular structure of (I), showing the atomic numbering scheme and displacement ellipsoids drawn at the 50% probability level.

**Fig. 2.**
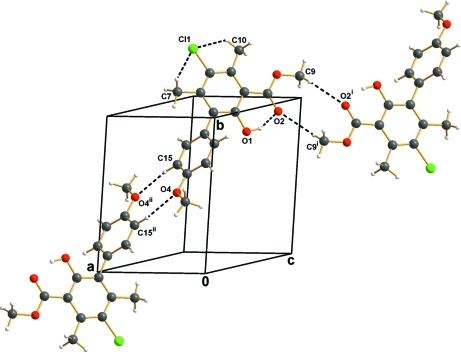
Part of the packing diagram of (I). Unique O—H···O, C—H···O and C—H···Cl interactions represented by dashed lines are shown.

## supplementary crystallographic information

### Comment

Functionalized biaryls containing a 3-arylsalicylate substructure occur in a variety of pharmacologically relevant natural products. The simple biaryls cynandione A—C have been isolated from many plant sources and show a considerable *in vitro* activity against hepatocytes, human bladder carcinoma T-24 cells, epidermoid carcinoma KB cells, and human hepatoma PLC/PRF/5 cells. For data on the pharmacological relevance of 3-arylsalicylates, see: Buchanan *et al.*, (1997), Huang *et al.*, (1999), Lin, Lin & Kuo (1997) and Lin, Wu & Kuo (1997). The sterically encumbered and functionalized biaryl, the title compound (I), was synthesized from 4-(4-methoxyphenyl)-1,3-bis(trimethylsilyloxy)-1,3-butadiene which is not readily available by other methods. In this paper, the crystal structure of (I) has been presented.

In the title compound (Fig. 1), the the dihedral angle between the mean planes of the two benzene rings is 65.92 (5)°. The methoxy group and the methylester group lie within the planes of the benzene rings to which they are bonded (deviation from mean planes: O2, -0.051 (2); O3, 0.151 (2); (??), 0.143 (2) Å; the torsion angles are: C2—C3—C8—O2 -174.47 (12) and C17—O4—C14—C15 -176.38 (12)°). There is an intramolecular hydrogen bond between the hydroxyl group and the carbonyl O atom of the methylester group. There are weak intramolecular interactions of the types C—H···O between atom O3 of the ester group and the adjacent methyl group (C10) and C—H···Cl between Cl1 and the adjacent methyl groups (C7/C10).

In the crystal structure, the molecules of (I) are held together by rather weak intermolecular C—H···O type non-classical hydrogen bonds resulting in dimeric units about inversion centers, forming eight and ten membered ring systems which may be described in terms of graph set notations (Bernstein *et al.* 1994) as R_2_^2^(8) and R_2_^2^(10) motifs for the hydrogen bonds: C15–H15···O4^ii^ and C9–H9A···O2^i^, respectively (details are given in Table 1 and Figure 2); leading to a zigzag chain arrangement.

### Experimental

The title compound was prepared according to a previously published procedure (Adeel *et al.*, 2009) using 3-chloro-4-trimethylsiloxy-pent-3-en-2-one (450 mg, 2.2 mmol), 4-(4-methoxyphenyl)-1,3-bis(silyloxy)-1,3-diene (806 mg, 2.2 mmol), and TiCl_4_ (0.241 ml, 2.2 mmol). (I) was isolated as a colourless crystalline solid. Re-crystallization from a saturated dichloromethane/methanol (9:1) solution at ambient temperature gave colourless crystals suitable for crystallographic studies.

### Refinement

The H atom bonded to O1 was located in a difference map and refined freely. Other H atoms were positioned geometrically and refined using a riding model, with C—H = 0.98 (methyl groups) or 0.95 Å (aryl CH) and with *U*_iso_(H) = 1.5 times *U*_eq_(C) (methyl groups) or with *U*_iso_(H) = 1.2 times *U*_eq_(C) (aryl CH). Torsion angles of all methyl groups were allowed to refine.

### Crystal data

**Table:**

C_17_H_17_ClO_4_	*Z* = 2
*M**_r_* = 320.76	*F*(000) = 336
Triclinic, *P*1	*D*_x_ = 1.388 Mg m^−^^3^
Hall symbol: -P 1	Melting point: 367 K
*a* = 6.534 (4) Å	Mo *K*α radiation, λ = 0.71073 Å
*b* = 9.574 (6) Å	Cell parameters from 7750 reflections
*c* = 12.694 (8) Å	θ = 6.4–59.5°
α = 97.420 (15)°	µ = 0.26 mm^−^^1^
β = 100.56 (2)°	*T* = 173 K
γ = 96.042 (14)°	Plate, colourless
*V* = 767.3 (8) Å^3^	0.55 × 0.27 × 0.01 mm

### Data collection

**Table:**

Bruker APEXII CCD diffractometer	3964 independent reflections
Radiation source: sealed tube	3091 reflections with *I* > 2σ(*I*)
graphite	*R*_int_ = 0.024
ω scans	θ_max_ = 29.0°, θ_min_ = 4.4°
Absorption correction: multi-scan (*SADABS*; Sheldrick, 2004)	*h* = −8→8
*T*_min_ = 0.868, *T*_max_ = 0.997	*k* = −13→13
14947 measured reflections	*l* = −17→17

### Refinement

**Table:**

Refinement on *F*^2^	Primary atom site location: structure-invariant direct methods
Least-squares matrix: full	Secondary atom site location: difference Fourier map
*R*[*F*^2^ > 2σ(*F*^2^)] = 0.040	Hydrogen site location: inferred from neighbouring sites
*wR*(*F*^2^) = 0.125	H atoms treated by a mixture of independent and constrained refinement
*S* = 1.09	*w* = 1/[σ^2^(*F*_o_^2^) + (0.073*P*)^2^ + 0.0592*P*] where *P* = (*F*_o_^2^ + 2*F*_c_^2^)/3
3964 reflections	(Δ/σ)_max_ < 0.001
207 parameters	Δρ_max_ = 0.31 e Å^−^^3^
0 restraints	Δρ_min_ = −0.23 e Å^−^^3^

### Special details

**Table:**

Experimental. Yield: 241 mg, 38%. m.p. = 367 (2) K. ^1^H NMR (250 MHz, CDCl_3_): δ = 2.10 (s, 3H, CH_3_), 2.56 (s, 3H, CH_3_), 3.77 (s, 3H, OCH_3_), 3.89 (s, 3H, OCH_3_), 6.89 (d, 2H, J = 8.8 Hz, ArH), 7.03 (d, 2H, J = 8.8 Hz, ArH), 10.54 (s, 1 H, OH). ^13^C NMR (62 MHz, CDCl_3_): δ = 19.0, 19.4 (CH_3_), 51.4, 54.2 (OCH_3_), 111.5 (C), 112.9 (2 C, CH), 126.8, 127.6, 128.3 (C), 129.9 (2 C, CH), 135.3, 140.8, 156.1, 157.8 (C), 170.5 (C=O). IR (KBr, cm ^-1^): ~ν = 3430 (*m*), 3050 (w), 3002 (w), 2959 (*m*), 2931 (*m*), 2837 (*m*), 1653 (*s*), 1607 (*m*), 1572 (w), 1514 (*s*), 1444 (*s*), 1373 (*m*), 1361 (*s*), 1297 (*s*), 1253 (*s*), 1220 (*s*), 1176 (*m*), 1092 (*m*), 1036 (*m*) 810 (*m*), 686 (*m*). GC—MS (EI, 70 eV): m/*z* (%): 322 (*M*^+^, ^37^Cl, 16), 320 (*M*^+^, 47), 288 (100), 260 (11), 245 (27), 225 (29), 181 (7), 152 (12). HRMS (EI, 70 eV): calcd for C_17_H_17_O_4_Cl [*M*, ^35^Cl]: 320.08099; found 320.08088.
Geometry. All s.u.'s (except the s.u. in the dihedral angle between two l.s. planes) are estimated using the full covariance matrix. The cell s.u.'s are taken into account individually in the estimation of s.u.'s in distances, angles and torsion angles; correlations between s.u.'s in cell parameters are only used when they are defined by crystal symmetry. An approximate (isotropic) treatment of cell s.u.'s is used for estimating s.u.'s involving l.s. planes.Least-squares planes (*x*,*y*,*z* in crystal coordinates) and deviations from them (* indicates atom used to define plane)- 3.7416 (0.0035) *x* + 4.8249 (0.0051) *y* - 7.9961 (0.0069) *z* = 2.4746 (0.0052)* 0.0019 (0.0009) C1 * -0.0098 (0.0009) C2 * 0.0101 (0.0008) C3 * -0.0023 (0.0008) C4 * -0.0059 (0.0008) C5 * 0.0061 (0.0008) C6 0.0307 (0.0018) C8 - 0.0505 (0.0021) O2 0.1508 (0.0021) O3 0.1865 (0.0030) C9Rms deviation of fitted atoms = 0.00683.1094 (0.0040) *x* + 6.1432 (0.0055) *y* - 9.0638 (0.0075) *z* = 4.7524 (0.0041)Angle to previous plane (with approximate su) = 65.92 (0.05)* 0.0117 (0.0009) C11 * -0.0089 (0.0009) C12 * -0.0016 (0.0009) C13 * 0.0091 (0.0009) C14 * -0.0060 (0.0010) C15 * -0.0043 (0.0010) C16 0.0476 (0.0018) O4 0.1434 (0.0024) C17Rms deviation of fitted atoms = 0.0077
Refinement. Refinement of *F*^2^ against ALL reflections. The weighted *R*-factor *wR* and goodness of fit *S* are based on *F*^2^, conventional *R*-factors *R* are based on *F*, with *F* set to zero for negative *F*^2^. The threshold expression of *F*^2^ > 2σ(*F*^2^) is used only for calculating *R*-factors(gt) *etc*. and is not relevant to the choice of reflections for refinement. *R*-factors based on *F*^2^ are statistically about twice as large as those based on *F*, and *R*- factors based on ALL data will be even larger.

### Fractional atomic coordinates and isotropic or equivalent isotropic displacement parameters (Å^2^)

**Table:**

	*x*	*y*	*z*	*U*_iso_*/*U*_eq_	
O1	−0.09153 (15)	0.87698 (10)	0.26779 (8)	0.0377 (2)	
H1	−0.175 (3)	0.892 (2)	0.3196 (17)	0.067 (6)*	
O2	−0.25708 (18)	0.99014 (12)	0.41459 (9)	0.0505 (3)	
O3	−0.16796 (18)	1.21628 (12)	0.48417 (9)	0.0491 (3)	
O4	0.23627 (16)	0.52864 (10)	−0.09022 (8)	0.0405 (2)	
Cl1	0.48249 (6)	1.39934 (4)	0.30599 (3)	0.05110 (15)	
C1	0.3067 (2)	1.24545 (14)	0.29830 (10)	0.0313 (3)	
C2	0.1636 (2)	1.24546 (14)	0.36668 (10)	0.0310 (3)	
C3	0.02086 (19)	1.11978 (13)	0.35519 (9)	0.0279 (3)	
C4	0.03415 (19)	1.00141 (13)	0.27908 (10)	0.0272 (3)	
C5	0.18323 (18)	1.00544 (13)	0.21221 (9)	0.0265 (3)	
C6	0.32076 (19)	1.12961 (13)	0.22127 (9)	0.0282 (3)	
C7	0.4785 (2)	1.14192 (16)	0.14895 (11)	0.0386 (3)	
H7A	0.4538	1.0572	0.0937	0.058*	
H7B	0.6207	1.1501	0.1925	0.058*	
H7C	0.4637	1.2265	0.1136	0.058*	
C8	−0.1451 (2)	1.10217 (14)	0.41965 (10)	0.0318 (3)	
C9	−0.3311 (3)	1.20118 (19)	0.54694 (14)	0.0515 (4)	
H9A	−0.4673	1.1710	0.4978	0.077*	
H9B	−0.3346	1.2925	0.5910	0.077*	
H9C	−0.3018	1.1298	0.5945	0.077*	
C10	0.1683 (3)	1.37309 (18)	0.45028 (13)	0.0513 (4)	
H10A	0.2995	1.4367	0.4576	0.077*	
H10B	0.1599	1.3421	0.5202	0.077*	
H10C	0.0487	1.4235	0.4275	0.077*	
C11	0.18957 (19)	0.87662 (13)	0.13357 (9)	0.0277 (3)	
C12	0.0254 (2)	0.82784 (14)	0.04645 (10)	0.0310 (3)	
H12	−0.0962	0.8750	0.0389	0.037*	
C13	0.0333 (2)	0.71183 (14)	−0.03026 (10)	0.0320 (3)	
H13	−0.0805	0.6811	−0.0899	0.038*	
C14	0.2094 (2)	0.64160 (13)	−0.01864 (10)	0.0312 (3)	
C15	0.3732 (2)	0.68633 (15)	0.06955 (11)	0.0366 (3)	
H15	0.4927	0.6372	0.0785	0.044*	
C16	0.3628 (2)	0.80242 (15)	0.14448 (11)	0.0348 (3)	
H16	0.4760	0.8322	0.2046	0.042*	
C17	0.0787 (3)	0.48428 (16)	−0.18492 (11)	0.0427 (3)	
H17A	−0.0537	0.4516	−0.1644	0.064*	
H17B	0.1213	0.4064	−0.2306	0.064*	
H17C	0.0596	0.5642	−0.2252	0.064*	

### Atomic displacement parameters (Å^2^)

**Table:**

	*U*^11^	*U*^22^	*U*^33^	*U*^12^	*U*^13^	*U*^23^
O1	0.0325 (5)	0.0332 (5)	0.0465 (5)	−0.0037 (4)	0.0149 (4)	−0.0005 (4)
O2	0.0459 (6)	0.0494 (7)	0.0592 (7)	−0.0045 (5)	0.0307 (5)	−0.0012 (5)
O3	0.0522 (7)	0.0473 (6)	0.0545 (6)	0.0061 (5)	0.0337 (5)	−0.0003 (5)
O4	0.0479 (6)	0.0350 (5)	0.0402 (5)	0.0133 (4)	0.0143 (4)	−0.0022 (4)
Cl1	0.0526 (3)	0.0427 (2)	0.0526 (2)	−0.01755 (17)	0.02060 (18)	−0.00782 (16)
C1	0.0298 (6)	0.0316 (7)	0.0294 (6)	−0.0037 (5)	0.0050 (5)	0.0013 (5)
C2	0.0305 (7)	0.0336 (7)	0.0270 (6)	0.0029 (5)	0.0054 (5)	−0.0007 (5)
C3	0.0247 (6)	0.0327 (6)	0.0268 (6)	0.0058 (5)	0.0056 (5)	0.0045 (5)
C4	0.0221 (6)	0.0296 (6)	0.0289 (6)	0.0029 (5)	0.0032 (4)	0.0039 (4)
C5	0.0236 (6)	0.0299 (6)	0.0256 (5)	0.0056 (5)	0.0030 (4)	0.0034 (4)
C6	0.0246 (6)	0.0345 (7)	0.0246 (5)	0.0022 (5)	0.0042 (4)	0.0040 (5)
C7	0.0358 (7)	0.0452 (8)	0.0354 (7)	−0.0010 (6)	0.0149 (6)	0.0024 (6)
C8	0.0274 (6)	0.0408 (7)	0.0293 (6)	0.0080 (6)	0.0074 (5)	0.0075 (5)
C9	0.0509 (9)	0.0617 (11)	0.0531 (9)	0.0171 (8)	0.0336 (8)	0.0092 (7)
C10	0.0572 (10)	0.0446 (9)	0.0485 (8)	−0.0076 (7)	0.0249 (7)	−0.0150 (7)
C11	0.0268 (6)	0.0287 (6)	0.0282 (6)	0.0045 (5)	0.0072 (5)	0.0032 (5)
C12	0.0291 (6)	0.0308 (6)	0.0325 (6)	0.0086 (5)	0.0040 (5)	0.0023 (5)
C13	0.0337 (7)	0.0318 (7)	0.0292 (6)	0.0058 (5)	0.0038 (5)	0.0016 (5)
C14	0.0370 (7)	0.0267 (6)	0.0336 (6)	0.0071 (5)	0.0152 (5)	0.0046 (5)
C15	0.0317 (7)	0.0418 (8)	0.0401 (7)	0.0154 (6)	0.0108 (6)	0.0061 (6)
C16	0.0274 (7)	0.0412 (8)	0.0348 (6)	0.0082 (6)	0.0038 (5)	0.0027 (5)
C17	0.0569 (9)	0.0345 (7)	0.0368 (7)	0.0054 (6)	0.0154 (6)	−0.0030 (5)

### Geometric parameters (Å, °)

**Table:**

O1—C4	1.3495 (17)	C7—H7C	0.9800
O1—H1	0.94 (2)	C9—H9A	0.9800
O2—C8	1.2205 (18)	C9—H9B	0.9800
O3—C8	1.3170 (18)	C9—H9C	0.9800
O3—C9	1.4496 (19)	C10—H10A	0.9800
O4—C14	1.3678 (16)	C10—H10B	0.9800
O4—C17	1.4169 (19)	C10—H10C	0.9800
Cl1—C1	1.7510 (16)	C11—C12	1.3850 (18)
C1—C2	1.3870 (19)	C11—C16	1.3937 (19)
C1—C6	1.4033 (18)	C12—C13	1.3914 (18)
C2—C3	1.417 (2)	C12—H12	0.9500
C2—C10	1.505 (2)	C13—C14	1.3869 (19)
C3—C4	1.4121 (18)	C13—H13	0.9500
C3—C8	1.4821 (19)	C14—C15	1.387 (2)
C4—C5	1.4052 (18)	C15—C16	1.382 (2)
C5—C6	1.3913 (19)	C15—H15	0.9500
C5—C11	1.4925 (18)	C16—H16	0.9500
C6—C7	1.5061 (19)	C17—H17A	0.9800
C7—H7A	0.9800	C17—H17B	0.9800
C7—H7B	0.9800	C17—H17C	0.9800
			
C4—O1—H1	104.4 (13)	O3—C9—H9C	109.5
C8—O3—C9	116.73 (12)	H9A—C9—H9C	109.5
C14—O4—C17	118.03 (11)	H9B—C9—H9C	109.5
C2—C1—C6	124.48 (12)	C2—C10—H10A	109.5
C2—C1—Cl1	118.94 (10)	C2—C10—H10B	109.5
C6—C1—Cl1	116.58 (10)	H10A—C10—H10B	109.5
C1—C2—C3	116.76 (12)	C2—C10—H10C	109.5
C1—C2—C10	120.28 (13)	H10A—C10—H10C	109.5
C3—C2—C10	122.94 (12)	H10B—C10—H10C	109.5
C4—C3—C2	119.62 (12)	C12—C11—C16	117.68 (12)
C4—C3—C8	116.31 (12)	C12—C11—C5	121.30 (11)
C2—C3—C8	124.06 (12)	C16—C11—C5	121.01 (11)
O1—C4—C5	115.99 (11)	C11—C12—C13	121.91 (12)
O1—C4—C3	122.28 (12)	C11—C12—H12	119.0
C5—C4—C3	121.72 (12)	C13—C12—H12	119.0
C6—C5—C4	118.97 (11)	C14—C13—C12	119.20 (12)
C6—C5—C11	121.90 (11)	C14—C13—H13	120.4
C4—C5—C11	119.13 (11)	C12—C13—H13	120.4
C5—C6—C1	118.42 (12)	O4—C14—C15	115.94 (12)
C5—C6—C7	121.32 (12)	O4—C14—C13	124.23 (12)
C1—C6—C7	120.25 (12)	C15—C14—C13	119.83 (12)
C6—C7—H7A	109.5	C16—C15—C14	120.05 (12)
C6—C7—H7B	109.5	C16—C15—H15	120.0
H7A—C7—H7B	109.5	C14—C15—H15	120.0
C6—C7—H7C	109.5	C15—C16—C11	121.29 (12)
H7A—C7—H7C	109.5	C15—C16—H16	119.4
H7B—C7—H7C	109.5	C11—C16—H16	119.4
O2—C8—O3	120.57 (12)	O4—C17—H17A	109.5
O2—C8—C3	123.38 (12)	O4—C17—H17B	109.5
O3—C8—C3	116.05 (12)	H17A—C17—H17B	109.5
O3—C9—H9A	109.5	O4—C17—H17C	109.5
O3—C9—H9B	109.5	H17A—C17—H17C	109.5
H9A—C9—H9B	109.5	H17B—C17—H17C	109.5
			
C6—C1—C2—C3	−1.3 (2)	Cl1—C1—C6—C7	−1.06 (16)
Cl1—C1—C2—C3	178.26 (9)	C9—O3—C8—O2	−0.3 (2)
C6—C1—C2—C10	177.07 (13)	C9—O3—C8—C3	179.38 (12)
Cl1—C1—C2—C10	−3.41 (19)	C4—C3—C8—O2	4.94 (19)
C1—C2—C3—C4	1.98 (18)	C2—C3—C8—O2	−174.47 (12)
C10—C2—C3—C4	−176.30 (12)	C4—C3—C8—O3	−174.73 (10)
C1—C2—C3—C8	−178.63 (11)	C2—C3—C8—O3	5.86 (19)
C10—C2—C3—C8	3.1 (2)	C6—C5—C11—C12	−113.50 (15)
C2—C3—C4—O1	177.11 (11)	C4—C5—C11—C12	66.49 (17)
C8—C3—C4—O1	−2.32 (17)	C6—C5—C11—C16	65.53 (17)
C2—C3—C4—C5	−1.34 (18)	C4—C5—C11—C16	−114.48 (14)
C8—C3—C4—C5	179.22 (10)	C16—C11—C12—C13	−2.1 (2)
O1—C4—C5—C6	−178.70 (10)	C5—C11—C12—C13	176.99 (12)
C3—C4—C5—C6	−0.15 (18)	C11—C12—C13—C14	0.9 (2)
O1—C4—C5—C11	1.31 (16)	C17—O4—C14—C15	−176.38 (12)
C3—C4—C5—C11	179.86 (10)	C17—O4—C14—C13	2.97 (19)
C4—C5—C6—C1	0.91 (17)	C12—C13—C14—O4	−178.45 (11)
C11—C5—C6—C1	−179.10 (10)	C12—C13—C14—C15	0.87 (19)
C4—C5—C6—C7	−177.74 (11)	O4—C14—C15—C16	178.07 (12)
C11—C5—C6—C7	2.25 (18)	C13—C14—C15—C16	−1.3 (2)
C2—C1—C6—C5	−0.19 (19)	C14—C15—C16—C11	0.0 (2)
Cl1—C1—C6—C5	−179.72 (9)	C12—C11—C16—C15	1.6 (2)
C2—C1—C6—C7	178.47 (12)	C5—C11—C16—C15	−177.44 (12)

### Hydrogen-bond geometry (Å, °)

**Table:**

*D*—H···*A*	*D*—H	H···*A*	*D*···*A*	*D*—H···*A*
O1—H1···O2	0.94 (2)	1.63 (2)	2.5061 (18)	153 (2)
C7—H7C···Cl1	0.98	2.74	2.957 (2)	93
C10—H10A···Cl1	0.98	2.45	3.003 (2)	115
C10—H10B···O3	0.98	2.28	2.662 (2)	102
C10—H10C···O3	0.98	2.57	2.662 (2)	85
C9—H9A···O2^i^	0.98	2.73	3.242 (3)	113
C9—H9C···O2^i^	0.98	2.96	3.242 (3)	98
C15—H15···O4^ii^	0.95	2.50	3.437 (2)	170

Symmetry codes: (i) −*x*−1, −*y*+2, −*z*+1; (ii) −*x*+1, −*y*+1, −*z*.
